# Supplementary material for: Atacama Large Aperture Submillimeter Telescope (AtLAST) science: The hidden circumgalactic medium
Source: Open Res Eur. 2024 Jun 13;4:117. [Version 1] doi: 10.12688/openreseurope.17452.1 (PMC11503815; doi:10.12688/openreseurope.17452.1)
Supplement: Peer review history

# Reviewer report: Reviewer response for version 1

**DOI:** 10.21956/openreseurope.18859.r44684

**Published:** 2024-10-23

**Authors:** Yongda Zhu

**Affiliations:** 1 University of Arizona, Tucson,, AZ, USA

This paper presents the scientific case for how the 50-m AtLAST will probe the circumgalactic medium (CGM). The CGM is critical to galaxy evolution, serving as a reservoir for gas accretion and a medium for feedback processes. Current sub-mm facilities struggle to detect the diffuse and extended cold gas components of the CGM. The paper reviews observational evidence, theoretical simulations, and technical challenges related to studying the cold CGM, spanning from the local to the high-redshift universe. It demonstrates how AtLAST will overcome these issues. The telescope's large aperture, high sensitivity, and wide field of view will enable detailed studies of the CGM across cosmic time. Its synergy with current and future facilities, such as ALMA, ngVLA, and X-ray missions, will allow for multi-phase CGM studies. The authors emphasize that AtLAST’s capability to detect cold gas tracers like [CII], CO, and [CI] across a wide field will have important implications for understanding the CGM and its role in galaxy evolution. Additionally, the paper outlines the technical and operational requirements for AtLAST.

 Overall, it is a well-written paper with intuitive figures and clear tables. The cartoon in Figure 1 is particularly helpful, and the comparison between mock ALMA observations vs. AtLAST observations is impressive. I recommend approval of this paper. However, I have some minor suggestions that the authors may consider including where they see fit.

 In the abstract, the authors explain the importance of the cold CGM but could improve clarity by briefly stating how AtLAST complements current sub-mm facilities like ALMA. This would provide immediate context for why AtLAST is needed.

 In the third paragraph of the introduction, the discussion about extended [CII] halos appears abruptly. To maintain flow and avoid confusion, the transition could be smoother by first introducing the broader context of CGM emission lines before moving to the specific case of [CII] halos.

 In Section "The missing baryon problem", Figure 2 shows a dip in baryon fraction curves, which isn’t explained in the text. The authors should explain—whether related to feedback mechanisms or observational limitations—so the dip doesn’t confuse readers.

 In the section "Impact of feedback from star formation and AGN", mentioning JWST NIRSpec/IFU may strengthen the discussion. JWST can help map gas kinematics at high redshift, complementing sub-mm and optical observations by resolving diffuse gas and providing direct kinematic data on CGM outflows and inflows (e.g., D'Eugenio et al., 2023)(Ref-1) .

 In Section 2.2 "gas-rich BCGs", instead of phrasing the statement as "These results are the opposite case of what is pictured...," consider discussing this as an environmental effect. This could lead to a better understanding of varying gas conditions in different environments.

 In Section 2.3, when mentioning that "such observations are extremely challenging for interferometers," it might be beneficial to explain why, and/or refer the reader to Section 4.1. Additionally, explaining "uv-coverage" and highlighting the need for short baselines would help general readers understand this challenge better.

 In Section 3.1, "Overlooked cold CGM and difficulties", it would be beneficial to mention that current simulations struggle to reproduce the observed evolution of metal absorbers in the high-z universe, particularly during the epoch of reionization (e.g., D’Odorico et al., 2022)(Ref-2), (Davies R et al.,2023)(Ref-3), (Becker et al., 2019)(Ref-4). This is relevant because metal absorption lines in quasar spectra are important for constraining the enrichment of high-z CGM.

 Also, consider including the work by (Stern et al., 2021)(Ref-5) with the FIRE simulation in this section.

 In Section 3.2, "Theoretical efforts towards AtLAST observations", where AGN feedback and molecular gas emission are still missing in the models, consider noting that AtLAST observations could help improve these models by providing additional constraints on these processes.

 In (IV) instrumental requirements, explain what baseline instability is and potential techniques to reduce it.

 In (V) a note on continuum observations, explain why current ALMA is not optimal for observing extended continuum emission compared to single-dish observations with AtLAST.

 In synergy with other facilities, consider adding a section discussing how JWST IFU could contribute to detecting diffuse gas kinematics and connecting to CGM studies.

 Some nitpicks:

 In Section 1.1.II, last paragraph: "namely in the CGM" -> "namely in the CGM (and IGM)"

 In Section 2.1, second paragraph: "tidal tail dwarf galaxies" -> "tidal dwarf galaxies."

 In Section 2.1, fourth paragraph: "the luminous infrared galaxy [,] and quasar host..." remove the comma.

 In Figure 6, some text from CASA is too small to read. Consider enlarging the text or removing the very small legends.

 In Figure 7, consider including the input image for a better comparison.

 In Table 2, although already clear, it could be helpful to include the frequency range in addition to mentioning “ALMA band 1 to 10.” For RMS and absolute flux calibration, adding some justification in the text could be helpful.

 Again, this is a very nice work!

Is the case presented with sufficient detail to be useful for teaching or other practitioners?

Yes

Is the work clearly and accurately presented and does it cite the current literature?

Yes

If applicable, is the statistical analysis and its interpretation appropriate?

Not applicable

Are all the source data underlying the results available to ensure full reproducibility?

Not applicable

Are the conclusions drawn adequately supported by the results?

Yes

Is the background of the case’s history and progression described in sufficient detail?

Yes

Reviewer Expertise:

Galaxy formation and evolution; reionization; intergalactic medium; optical/NIR/sub-mm observations of high-z galaxies/quasars.

I confirm that I have read this submission and believe that I have an appropriate level of expertise to confirm that it is of an acceptable scientific standard.

## References

[1] : A fast-rotator post-starburst galaxy quenched by supermassive black-hole feedback at z . *Nature Astronomy* .2024; 10.1038/s41550-024-02345-1 10.1038/s41550-024-02345-1

[2] : The evolution of the Si iv content in the Universe from the epoch of reionization to cosmic noon. *Monthly Notices of the Royal Astronomical Society* .2022;512(2) : 10.1093/mnras/stac545 2389-2401 10.1093/mnras/stac545

[3] : Examining the decline in the C iv content of the Universe over 4.3 ≲ z ≲ 6.3 using the E-XQR-30 sample. *Monthly Notices of the Royal Astronomical Society* .2023;521(1) : 10.1093/mnras/stad294 314-331 10.1093/mnras/stad294

[4] : The Evolution of O i over 3.2 < z < 6.5: Reionization of the Circumgalactic Medium. *The Astrophysical Journal* .2019;883(2) : 10.3847/1538-4357/ab3eb5 10.3847/1538-4357/ab3eb5

[5] : Virialization of the Inner CGM in the FIRE Simulations and Implications for Galaxy Disks, Star Formation, and Feedback. *The Astrophysical Journal* .2021;911(2) : 10.3847/1538-4357/abd776 10.3847/1538-4357/abd776

# Reviewer report: Reviewer response for version 1

**DOI:** 10.21956/openreseurope.18859.r44677

**Published:** 2024-10-22

**Authors:** David T. Frayer

**Affiliations:** 1 Green Bank Observatory, Green Bank, WV, USA

Overview:

 The authors present a case study for AtLAST, a proposed future telescope, focusing on observations of the extended cold gas component within the circumgalactic medium (CGM) and integalactic medium (IGM). This paper is part of a series of white papers that outline the scientific advantages of AtLAST.     The authors provide an overview of CGM science, incorporating both observational and theoretical reviews.  The paper show simulations to highlight what AtLAST could accomplish in comparison to the current ALMA facility for imaging a z=6.5 star-forming galaxy system and its evolutional counterpart at z=0.01.  The authors also discuss the synergies of AtLAST with other facilities for studies of the CGM.

 The paper highlights the need of a large single dish to provide sensitive measurements of low surface brightness emission over large areas of the sky.  The science presented here cannot be fully realized by using only interferometers, such as ALMA, and the total power measurements associated with ALMA are woefully inadequate in comparison to AtLAST.  A large single dish provides better sensitivity for total power observations than the current ALMA-TP+ACA approach. This paper highlights the importance of a large single-dish operating in the sub-mm for the study of the CGM, for both the local universe and in the early universe during the formative phases of galaxies and clusters of galaxies.

 Suggestions:

 The authors could provide more theoretical context on why cold flows are expected at high-reshift (e.g., senarios C,D of Fig.1), while at low redshift hot gas accretion dominates (e.g., senarios A,B of Fig. 1). Due to the lower mass of the dark-matter halos at high-z, the cold gas streaming along filaments can penetrate the hot halo to feed the central star-forming regions (e.g,. Dekel & Birnboim 2006),

 while the more massive halos at low-z result in hot-mode accretion (e.g., Keres+2005).  One of the most interesting science questions addressed by AtLAST would be characterizing the transition phase between cold-flow and hot-flow accretion in the evolution of galaxies.

 In Figure 6, the resolution and the rms of the images are not presented for the ALMA CASA simulation.

 For Figure 7, one should compare data at similar resolutions (currently comparing 0.5" ALMA vs 2" AtLAST).  Recommend using a lower-resolution ALMA configuration for the simulation (e.g., C3) and then smoothing/tappering the ALMA data to match the AtLAST resolution. A simulation showing ALMA combined with ALMA-TP and/or ALMA-ACA data compared to AtLAST would also help to make a compelling case.

 Comments:

 A 50m telescope operating in the sub-millimeter range will pose significant technical challenges in maintaining the necessary surface, pointing, and focus accuracy.  The approaches developed for AtLAST may also be applicable to help enhance the operation of other large single-dish facilities.

Is the case presented with sufficient detail to be useful for teaching or other practitioners?

Yes

Is the work clearly and accurately presented and does it cite the current literature?

Yes

If applicable, is the statistical analysis and its interpretation appropriate?

Not applicable

Are all the source data underlying the results available to ensure full reproducibility?

Not applicable

Are the conclusions drawn adequately supported by the results?

Yes

Is the background of the case’s history and progression described in sufficient detail?

Yes

Reviewer Expertise:

Extragalactic radio/mm/sub-mm/IR observations of gas-rich infrared luminous galaxies at high-redshift and the local universe.

I confirm that I have read this submission and believe that I have an appropriate level of expertise to confirm that it is of an acceptable scientific standard.
